# Variability in adherence to clinical practice guidelines and recommendations in COPD outpatients: a multi-level, cross-sectional analysis of the EPOCONSUL study

**DOI:** 10.1186/s12931-017-0685-8

**Published:** 2017-12-02

**Authors:** Myriam Calle Rubio, José Luis López-Campos, Juan J. Soler-Cataluña, Bernardino Alcázar Navarrete, Joan B. Soriano, José Miguel Rodríguez González-Moro, Manuel E. Fuentes Ferrer, Juan Luis Rodríguez Hermosa, On behalf of the EPOCONSUL Study, Jose Luis Rojas Box, Jose Domingo Garcia Jimenez, Adolfo Domenech del Rio, Ana Muñoz, Antonia Soto Venegas, Aurelio Arnedillo Muñoz, Agustín Valido Morales, Jose Velasco Garrido, Carlos Rueda Ríos, Macarena Arroyo Varela, Francisco Ortega Ruiz, Eduardo Marquez Martin, Carmen Calero Acuña, Francisco Luis Garcia Gil, Joaquin Carlos Costan Galicia, Ana Boldova Loscertales, Cristina Martinez González, Rosirys Guzman Taveras, Juan Luis De la Torre Alvaro, Mª. Jesus Avilés Ingles, Rubén Andújar Espinosa, Juan Manuel Palmero Tejera, Juan Marco Figueira Conçalves, Ramon Agüero Balbín, Carlos Amado Diago, Beatriz Abascal Bolado, Juan Luis Garcia Rivero, Marcelle Cohen Escovar, Francisco Javier Callejas González, Angel Ortega Gonzalez, Rosario Vargas Gonzalez, Encarnación López Gabaldón, Raul Hidalgo Carvajal, Elena Bollo de Miguel, Silvia Fernández Huerga, Ana Pueyo Bastida, Jesus R. Hernández Hernández, Ruth Garcia García, Miguel Barrueco Ferrero, Marco López Zuibizarreta, E. Consuelo Fernández, David De la Rosa Carrillo, Jordi Esplugas Abós, Noelia Pablos Mateos, Elena De Miguel Campos, Pablo Rubinstein, Hernán Abraham Manrique Chávez, Miriam Barrecheguren, Carmen Aguar Benito, Pablo Catalán Serra, Eusebi Chiner Vives, Juan Antonio Royo Prats, Cristina Sabater Abad, Esther Verdejo Mengual, Eva Martínez-Moragon, Francisca Lourdes Marquez Perez, Alberto Fernandez Villar, Cristina Represas Represas, Ana Priegue Carrera, Marina Blanco Aparicio, Pedro Jorge Marcos Rodriguez, Federico Gonzalo Fiorentino, Mª. Magdalena Pan Naranjo, Antonia Fuster Gomila, German Peces Barba, Felipe Villar Alvarez, Carlos Jose Álvarez Martinez, Juan Luis Rodriguez Hermosa, J. L. Álvarez Sala-Walther, Juan Rigüal Bobillo, Gianna Vargas Centanaro, José Andrés García Romero de Tejada, Javier Jareño, Sergio Campos Tellez, Raul Galera Martinez, Rosa Mar Gómez Punter, Emma Vázquez Espinosa, Esther Alonso Peces, Juan Manuel Diez Piña, Raquel Pérez Rojo, Luis Puente Maestu, Julia Garcia de Pedro, Soledad Alonso Viteri, Maria Hernandez Bonaga, Maria Milagros Iriberri Pascual, Myriam Aburto Barrenechea, Sophe Garcia Fuika, Patricia Sobradillo Ecenarro

**Affiliations:** 1 Pulmonary Department, Research Institute of Hospital Clínico San Carlos (IdISSC), Faculty of Medicine, University Complutense of Madrid, C/ Martin Lagos s/n, 28040 Madrid, Spain; 2 Unidad Médico-Quirúrgica de Enfermedades Respiratorias, Instituto de Biomedicina de Sevilla (IBiS), Hospital Universitario Virgen del Rocío/Universidad of Sevilla, Sevilla, Spain; 3 CIBER de Enfermedades Respiratorias (CIBERES), Instituto de Salud Carlos III, Madrid, Spain; 4 Pulmonary Department, Hospital of Arnau de Villanova, Valencia, Spain; 5 Pulmonary Department, Hospital of Alta Resolución de Loja, Granada, Spain; 6 Research Institute of Hospital University La Princesa (IISP), University Autónoma of Madrid, Madrid, Spain; 7 Pulmonary Department, H. Universitario Príncipe de Asturias, Alcalá de Henares, Madrid, Spain; 8 UGC de Medicina Preventiva, Research Institute of Hospital Clínico San Carlos (IdISSC), Hospital Clínico San Carlos, Faculty of Medicine, University Complutense of Madrid, Madrid, Spain

**Keywords:** Chronic obstructive pulmonary disease, Clinical audit, Medical care, Clinical practice guidelines, Adherence to recommendations

## Abstract

**Background:**

Clinical audits have reported considerable variability in COPD medical care and frequent inconsistencies with recommendations. The objectives of this study were to identify factors associated with a better adherence to clinical practice guidelines and to explore determinants of this variability at the the hospital level.

**Methods:**

EPOCONSUL is a Spanish nationwide clinical audit that evaluates the outpatient management of COPD. Multilevel logistic regression with two levels was performed to assess the relationships between individual and disease-related factors, as well as hospital characteristics.

**Results:**

A total of 4508 clinical records of COPD patients from 59 Spanish hospitals were evaluated. High variability was observed among hospitals in terms of medical care. Some of the patient’s characteristics (airflow obstruction, degree of dyspnea, exacerbation risk, presence of comorbidities), the hospital factors (size and respiratory nurses available) and treatment at a specialized COPD outpatient clinic were identified as factors associated with a better adherence to recommendations, although this only explains a small proportion of the total variance.

**Conclusion:**

To be treated at a specialized COPD outpatient clinic and some intrinsic patient characteristics were factors associated with a better adherence to guideline recommendations, although these variables were only explaining part of the high variability observed among hospitals in terms of COPD medical care.

## Background

Chronic obstructive pulmonary disease (COPD) is one of the most frequent reasons for seeking medical attention and accounts for 10% of primary care and 30% of outpatient respiratory care visits [1]. Patients with this condition have a high morbidity and mortality [2, 3]. For these reasons, there are a number of clinical practice guidelines (CPG) aimed to systemize medical care for COPD [4–7]. However, the real-life implementation of these CPG is low [8, 9].

Clinical audits have emerged as an overarching tool to measure the adequacy of clinical practice and feedback is being used to improve health care [10]. For more than 12 years, some countries have been auditing the quality of their in-hospital COPD management in a systematic way [11–13]. However, we have less evidence regarding outpatient care, and the few existing studies only explored certain aspects, such as the diagnosis or the prescription pattern, showing us outpatient care is far from perfect [14–18] with considerable variability in COPD medical care and frequent inconsistencies with CPG recommendations.

The EPOCONSUL study is the first national audit to evaluate the adequacy of medical care according to CPG in Spain in COPD patients treated at outpatient respiratory clinics. The study confirmed significant variations in adherence to CPG recommendations between centers [19]. The objective of our work has been analyze the variability and to identify factors associated with adherence to current recommendations for COPD clinical practice guidelines for outpatients in Spain.

## Methods

The methodology of the EPOCONSUL audit has been extensively described elsewhere [19]. Briefly, the COPD audit promoted by the Spanish Society of Pneumology and Thoracic Surgery (SEPAR) was designed to evaluate clinical practice as well as clinical and organizational factors related to managing patients with COPD across Spain. It was designed as an observational non-interventional cross-sectional study. Recruitment was intermittent during the year (May 2014–May 2015). Every 2 months each investigator recruited clinical records of the first 10 patients identified as diagnosed with COPD and seen in the outpatient respiratory clinic. Subsequently, patients identified were reevaluated to determine if they met the inclusion/exclusion criteria described in Appendix 1. The sampling process was detailed in an epidemiology flow chart and described in Appendix 2.

The information collected was historical in nature for the clinical data of the last and previous visits, and the information about hospital resources was concurrent.

As described in the methodological research paper [19], in order to evaluate the degree of current CPG implementation of the main statements according to the 2012 Spanish National Guidelines for COPD care (GesEPOC) and the 2013 Global initiative for chronic Obstructive Lung Disease (GOLD), we established fulfilling ≥ 50% of criteria for good clinical practice evaluated in each category (clinical evaluation of the patient, COPD evaluation and therapeutic intervention) as the cut-off point.

From the 175 public hospitals in the National Health System invited from the Spanish Society of Pneumology and Thoracic Surgery, 59 participated (33.3%). The estimated reference population for the EPOCONSUL study was 18,104,350 inhabitants, representing 39% of the Spanish population. The distribution of hospitals in the different regions and the population covered by those hospitals are detailed in Appendix 3 and participating investigators are included in Appendix 4.

In order to compare hospitals, these were divided in two types of center according to their size (small or large) as measured by: the number of beds per center ≥ 500, the number of inpatient respiratory beds ≥ 20, the number of pulmonology staff members ≥ 5, and the number of annual outpatient respiratory visits ≥ 10,000. All the criteria are necessary to be considered large.

The protocol was approved by the Ethics Committee of the Hospital Clínico San Carlos (Madrid, Spain; internal code 14/030-E). Additionally, according to current research laws in Spain, the ethics committee at each participating hospital evaluated and agreed to the study protocol. The need for informed consent was waived because ours is a clinical audit, beside the non-interventional nature of the study, the anonymization of data and the need to blindly evaluate the clinical performance. This circumstance was clearly explained in the protocol, and the ethical committees approved this procedure. To avoid modifications to the usual clinical practice and preserve the blinding of the clinical performance evaluation, the medical staff responsible for the outpatient respiratory clinic was not informed about the audit. Data was entered remotely at each participating location to a centrally-controlled server.

### Statistical analysis

Descriptive results are presented both at the patient and hospital level. Qualitative variables were summarized by their frequency distribution as well as quantitative variables by their median, interquartile range (IQR) and minimum–maximum. The differences between hospital resources and characteristics according to size (small vs large) were evaluated using χ2 tests for categorical data, while the non-parametric Mann-Whitney test was used for continuous data. Significance of variability by area/hospital was explored by the Kruskal–Wallis or chi-square tests.

With regard to missing data, after performing data cleansing to identify and correct missing and extremely unlikely values, the data was included in the analysis as missing information.

Three dependent variables were generated to evaluate the degree of CPG implementation; criteria of good clinical practice were categorized into: fulfilling three criteria at the clinical evaluation, fulfilling four criteria at the COPD evaluation, and fulfilling three criteria at the therapeutic intervention.

The association between each independent variable (patient characteristics, hospital resources and work organization) and each of the dependent variables was assessed by calculating the crude odds ratio (OR) via multilevel bivariate regression analysis. Each multilevel analysis included two levels: the individual or patient level (level 1), and the hospital level (level 2). It was developed in four consecutive steps: (1) Model 1 (empty model) which included only the dependent variable and the hospital-cluster effect; (2) random effects Model 2, which included the hospital variables; (3) random effects Model 3, which included the patient variables; (4) random effects Model 4, which included the patient and hospital variables in order to obtain an overall multivariable model. Candidate predictors with a value of *p* < 0.10 in the univariate analysis were accepted for inclusion in the multivariate analysis in model 2 and 3. Variables were removed from the model when the *p*-value exceeded 0.10 and were kept in the final model when less than 0.05. The independent predictor variables included in Model 4 were those selected in the last step in Models 2 and 3. The coefficients of the predictor variables were transformed into OR, with 95% confidence intervals (CI).

The hospital cluster effect was evaluated and quantified by two indicators: 1) the intra-cluster correlation coefficient (ICC) which represents the proportion of the variance attributable to the clustering effect and 2) the median odds ratio (MOR). The MOR can be interpreted as the median increased odds of reaching the outcome if an individual was admitted to another hospital with a greater risk of that outcome.

All analyses were performed using STATA 12.0 software. Statistical significance was assumed as *p* < 0.05.

## Results

A total of 17,893 clinical records of patients treated in outpatient respiratory clinics were evaluated during the study period and 5726 clinical records of patients presumably diagnosed with COPD were selected. Of them, 4508 patients were audited from 59 hospitals, for having all the inclusion criteria and none of the exclusion criteria. The sampling process was detailed in an epidemiology flow chart and described in Appendix 2.

### Center characteristics

The hospital characteristics and respiratory unit resources are summarized in Table 1. Large hospitals constituted 54% of centers. The majority participating centers were public (93.2%), university hospitals (83.1%) and had a pulmonary resident available (67.8%). Although the larger hospitals had more staff, the length of the outpatient follow-up visit was similar. There were few centers with a specialized COPD outpatient clinic (47.5%) and outpatient respiratory nursing clinic (45.8%), regardless of hospital size.

**Table 1:** Characteristics of the participating hospitals and resources of the respiratory units

	All	Small hospital	Large hospital	*P* ^†^
Number of participating hospitals, *n*	59	27	32	
Public hospital (%)	93.2	85.2	100	0.039
University hospital (%)	83.1	63	100	< 0.001
Beds per center ≥ 500 (%)	62.7	18.5	100	< 0.001
Beds per center, median (P25–75)	651 (349–943)	332 (231–436)	903 (702–1199)	< 0.001
Hospital with a respiratory ward (%)	83.1	63	100	< 0.001
Number of inpatient respiratory beds ≥ 20 (%)	83.7	52.9	100	< 0.001
Number of pulmonology staff members ≥ 5 (%)	81.4	59.3	100	< 0.001
Number of pulmonology staff members, median (P25–75)	10 (5–13)	5 (2–8)	13 (10–16)	< 0.001
Pulmonology residents available (%)	67.8	33.3	96.9	< 0.001
Number of annual outpatient respiratory visits, median (IQR)	15,447 (12004–25,680)	12,004 (4355–13,556)	23,985 (16070–27,838)	< 0.001
Number of annual outpatient respiratory visits ≥ 10,000 (%)	81.4	59.3	100	< 0.001
≥ 15 min of follow-up at general outpatient respiratory visit (%)	44.1	48.1	40.6	0.562
Specialized COPD outpatient clinic available (%)	47.5	40.7	53.1	0.343
≥ 15 min of follow-up at specialized COPD outpatient visit (%)	64.4	74.1	56.3	0.154
Outpatient respiratory nursing clinic availability (%)	45.8	44.4	46.9	0.852
Functional respiratory laboratory available (%)				
- Spirometry	100	100	100	1
- Diffusing capacity	100	100	100	1
- Plethysmography	100	100	100	1
- Respiratory muscle strength	84.7	66.7	100	< 0.001
- 6MWT available	94.9	88.9	100	0.090
- Cardiopulmonary exercise testing available	62.7	40.7	81.3	0.001
Inhalation technique educational program available (%)	30.5	15.6	48.1	0.007
Respiratory rehabilitation program available (%)	74.6	66.7	81.3	0.2
- Hospital-based	61.4	61.1	61.5	0.617
- Home-based	6.8	11.1	3.8	
- Mixed	31.8	27.8	34.6	

Data are presented as median (CI 95%), unless stated otherwise. Dichotomous variables are expressed as number and/or percent. *p* ^†^ calculated by the Kruskal–Wallis or Chi-square test, depending on the nature of the variable

### Audited patient characteristics and clinical conditions

The main characteristics of the patients evaluated are presented in Table 2.

**Table 2:** Clinical characteristics of the audited cases

	Patients (*N* = 4508)		Hospitals (*N* = 59)		*p* ^†^
	*N*	% or median (IQR)	Median	IQR	
Sex (male), (%)	4.508	86	87.5	82.1–93.2	< 0.001
Age (years), median (P25–75)	4.508	69.7 (63–77.7)	70	69–72	< 0.001
≤ 55 (%)		8.5	8.2	5.8–11.7	
56–69 (%)		38.7	38.1	30–42.6	
≥ 70 (%)		52.8	53.3	47.1–61.7	
Pack-years, median (P25–75)	4.508	47 (34–70)	45	40–51	< 0.001
Active smokers, (%)	4.508	23.1	22	18–29	< 0.001
BMI kg/m2, median (P25–75)	4.508	28.0 (24.4–31.1)	27.8	26.6–28.5	0.03
≤ 21 (%)		7.1	6.7	4.1–9.2	
22–29 (%)		60.8	58.8	56.1–64	
≥ 30 (%)		32.1	31.4	26.2–37.7	
Charlson index, median (P25–75)	4.508	2 (1–4)	2	2–3	< 0.001
≥ 3 (%)		44.9	44.5	40–56.6	
Dyspnea (MRC-m)	4.508				< 0.001
0 + 1 (%)		27.3	23.8	11.6–44.5	
≥ 2 (%)		41.4	38.3	28.3–54	
Missing (%)		13.2	8.9	1.6–21.6	
Level of dyspnea not referred to (%)		18.1	11.6	3.3–30	
CAT questionnaire >10, (%)	869	62.4	64	47.9–83.8	< 0.001
Chronic bronchitis criteria, (%)	4.508	41.7	41	28.3–51	< 0.001
Chronic colonization, (%)	4.508	6.0	5	3.2–8.3	< 0.001
Symptoms suggestive of asthma,(%)	4.508	26.5	18.3	10.8–35	< 0.001
% FEV1, median (P25–75)	4.508	50 (37–63)	51	47–54	0.03
< 50%		49.1	45.5	41.5–53.3	
50–64%		28	28.5	22.3–31.7	
≥ 65%		22.9	23.8	15–30	
Number of moderate/severe exacerbations in the last year, median (P25–75)	3.196	1.1 (0–2)	1	0–1	0.03
Number of hospital admissions in the last year, median (P25–75)	4508	0.5 (0–1)	0	0–0	0.03
BODE value, median (P25–75)	632	3.9 (3–5)	4.5	3–5.5	< 0.001
GOLD group	985				< 0.001
A (%)		22.7	14.3	0–25.9	
B (%)		18.7	16.7	0–24.1	
C (%)		18.7	20	9.8–33.3	
D (%)		39.9	40	23.5–55.6	
GesEPOC Phenotype	4.508				< 0.001
- Non-exacerbator, (%)		27.5	24.4	11.4–28	
- Exacerbator, (%)		18.8	15.7	3.4–22	
- Missing, (%)		53.7	52.3	44–58.9	
LAMA monotherapy, (%)	4.391	10.0	10	4.8–15.3	0.03
LAMA-LABA combination, (%)	4.391	22.7	20.3	14.5–27.9	< 0.001
LABA+ ICS combination, (%)	4.391	7.7	6.7	3.4–9.8	0.03
Triple therapy (LAMA + LABA + CSI), (%)	4.391	49.1	50.8	39.3–60.3	< 0.001
Long-term oxygen therapy, (%)	4.508	26.6%	25	17.1–33.3	0.03
Home ventilation, (%)	4.508	7.5%	5	2.5–11.6	< 0.001
Respiratory rehabilitation, (%)	4.508	9	5	0–11.8	< 0.001
Respiratory care follow-up (years), (%)	4.508	4 (2–7)	4	3.5–5	0.03

Dichotomous variables are expressed as *n* and percentage. Average value expressed as median (P25–75). The variability was expressed using the interquartile range (IQR) of median. ^†^Calculated for the variability between centers using test de Kruskal–Wallis or chi-square test, depending on the nature of the variable

*Abbreviations*: *LABA* long-acting beta-2 agonists, *LAMA* long-acting antimuscarinic agents, *ICS* inhaled corticosteroids, *GOLD* Global Initiative for Chronic Obstructive Lung Disease, *GesEPOC* Spanish National Guidelines for COPD, *CAT* COPD Assessment Test

### Adequacy of medical care according to CPG

Adherence to the main CPG statements is summarized in Table 3. There was a significant variation between hospitals, with a better adherence to the statements in the clinical evaluation category, with three out of six criteria fulfilled in 65.5% of the patients.

**Table 3:** Adherence to recommendations (GOLD and GesEPOC) evaluated in the study and classified in three categories: clinical evaluation of the patient, COPD evaluation and therapeutic interventions. The number of criteria or quality standards fulfilled was analyzed in each category

Criteria of good clinical practice evaluated in EPOCONSUL	N of criteria fulfilled	Patients (*N* = 4.508) %	Hospitals (*N* = 59) Median	Inter-hospital range	*p* ^†^
Clinical evaluation category					
1. Was degree of dyspnea evaluated during current visit? 2. Was the number of hospital admissions in the last 12 months collected during current visit? 3. Was the number of moderate or severe exacerbations in the last 12 months collected during current visit? 4. Was current smoking habit information collected? 5. Was regular exercise data collected during current visit? 6. Are comorbidities identified in the clinical record?	6 criteria	18.3	14.6	0–100	< 0.001
	>3 criteria	65.5	70	11.7–100	< 0.001
	≤3 criteria	34.5	30	0–88.3	< 0.001
COPD evaluation category					
1. Alfa-1-antitrypsin serum level determination available? 2. COPD severity defined in the report? 3. COPD GOLD type defined in the report? 4. COPD phenotype according to GesEPOC defined in the report? 5. 6MWT carried out on any occasion? 6. Diffusion capacity measured on any occasion? 7. Lung volumes measured on any occasion? 8. Chest CT scan carried out on any occasion in exacerbator phenotype?	8 criteria	1.5	0	0–14.6	< 0.001
	> 4 criteria	30.1	27	0–89.3	< 0.001
	≤ 4 criteria	69.9	73	10.7–100	< 0.001
Therapeutic intervention category					
1. Is treatment adherence evaluated in any way? 2. Is inhalation technique evaluated in any way? 3. Is Pneumococcal vaccination recommended? 4. Is exercise advised during the visit? 5. Have arterial blood gases been measured on any occasion in patients on long-term oxygen therapy? 6. Is a specific intervention for smoking cessation for active smokers offered?	6 criteria	9.3	3.3	0–45.1	< 0.001
	> 3 criteria	22.4	12.5	0–100	< 0.001
	≤ 3 criteria	77.6	87.5	0–100	< 0.001

Dichotomous variables are expressed as *n* and percentage. The variability between centers was expressed using the inter-hospital range (min–max). *p* ^†^ was calculated for the variability between centers using the Kruskal–Wallis or Chi-square tests, depending on the nature of the variable

### Adherence to CPG recommendations based on patient and center characteristics

The bivariate association between adherence to the main CPG statements and the variables related to hospital and patient characteristics is summarized in Appendix 5. A major number of the patient-level variables were associated with adherence, whereas the majority of center-level variables were not.

### Multilevel variability analysis of adherence to CPG recommendations

For the adherence to the statements in the clinical evaluation category, fulfillment of at least three criteria, the percentage of the total variability attributable to the hospital-cluster effect was 36%. The empty model exhibited a significant cluster effect (ICC = 0.36) and cluster heterogeneity (MOR = 3.73). In the adjusted model, being an active smoker, having a Charlson index ≥ 3, undergoing ≥ 1 hospitalization for COPD in the past year and being treated at a specialized COPD outpatient clinic was positively associated. Only one variable linked to the hospital level (large hospital) was retained in the model as a predictor, but was unfavorable (Table 4). The inclusion of all predictors further reduced the residual between-hospital cluster variability. The ICC and MOR dropped to 0.31 and 3.26, respectively (Table 4). Some unrecorded values (COPD phenotype missing) showed significant associations, which is naturally open to interpretation.

**Table 4:** Multilevel logistic regression models of the variability in adherence to good clinical practice criteria for three categories: clinical evaluation of the patient, disease evaluation and therapeutic interventions

	Intra-class correlation (ICC)	Median Odds Ratio (MOR)	Variables	Adjusted OR (95% CI)	*p*
Adherence to good clinical practice criteria in clinical evaluation (≥3 criteria fulfilled)					
Empty model 1	0.36670	3.73040			
Model 2: center variables^1^	0.31866	3.26487			
Model 3: patient variables^2^	0.36831	3.74755			
Full model 4 (center and patient)	0.31850	3.26345	Large hospital	0.40 (0.21–0.79)	0.008
			Outpatient respiratory nursing clinic available	2.47 (1.26–4.83)	0.008
			Active smokers	1.32 (1.10–1.58)	0.003
			Charlson index ≥3	1.35 (1.15–1.59)	< 0.001
			Number of hospital admissions in the last year ≥1	6.33 (5.02–7.98)	< 0.001
			GesEPOC phenotype		
			Not exacerbator (reference)		
			Exacerbator	0.79 (0.61–1.101)	0.063
			Missing	0.36 (0.29–0.44)	< 0.001
			To be taken care in specialized COPD outpatient clinic)	2.10 (1.56–2.72)	< 0.001
1: included variables in the final center model: large hospital and outpatient respiratory nursing clinic available 2: included variables in the final patient model: active smokers, Charlson index ≥3, number of hospital admissions in the last year ≥1, to be taken care in specialized COPD outpatient clinic and GesEPOC phenotype exacerbator.					
Adherence to good clinical practice criteria in COPD evaluation (≥4 criteria fulfilling)					
Empty model 1	0.30343	3.13266			
Model 2: center variables^1^	0.26684	2.83994			
Model 3: patient variables^2^	0.29100	3.02953			
Full model 4 (center and patient)	0.24413	2.67316	Respiratory ward not available (reference)		
			Respiratory ward < 20 beds	7.09 (2.53–9.90)	< 0.001
			Respiratory ward ≥20 beds	3.00 (1.37–6.60)	0.006
			Age ≤ 55	1.58 (1.19–2.09)	0.001
			Sex (male)	0.77 (0.61–0.96)	0.022
			Charlson index ≥3	0.80 (0.68–0.94)	0.008
			FEV1 < 50%	1.68 (1.42–1.99)	< 0.001
			Dyspnea (MRC-m)		
			0–1 (reference)		
			≥ 2	1.39 (1.13–1.72)	0.002
			Missing	0.69 (0.51–0.93)	0.017
			Level of dyspnea not referred to	0.65 (0.49–0.86)	0.003
			GesEPOC phenotype		
			Non-exacerbator (reference)		
			Exacerbator	1.16 (0.93–1.44)	0.185
			Missing	0.17 (0.14–0.21)	< 0.001
			Treatment at a specialized COPD outpatient clinic	3.25 (2.49–4.23)	< 0.001
1: variables included in the final center model: in-patient respiratory clinic ≥20 present and specialized COPD outpatient clinic available. 2: variables included in the final patient model: age ≤ 55, gender (male), Charlson index ≥3, FEV1 < 50%, dyspnea, GesEPOC exacerbator phenotype and being treated in specialized COPD outpatient clinic.					
Adherence to good clinical practice criteria in therapeutic intervention (≥3 criteria fulfilled)					
Empty model 1	0.52169	6.09155			
Model 2: center variables^1^	0.46935	5.08927			
Model 3: patient variables^2^	0.49994	5.64024			
Full model 4 (center and patient)	0.44731	4.74211	University hospital	0.26 (0.08–0.85)	0.026
			Outpatient respiratory nursing clinic availability	3.69 (1.50–9.11)	0.005
			Age ≤ 55	0.60 (0.42–0.86)	< 0.005
			Sex (male)	0.72 (0.55–0.93)	0.014
			Charlson index ≥3	1.19 (0.99–1.42)	0.062
			Number of hospital admissions in the last year ≥1	1.71 (1.38–2.11)	< 0.001
			GesEPOC phenotype		
			Non-exacerbator (reference)		
			Exacerbator	0.90 (0.71–1.15)	0.404
			Missing	0.36 (0.29–0.46)	< 0.001
			Treatment at a specialized COPD outpatient clinic	2.61 (2.01–3.40)	<0.001
1: variables included in the final center model: university hospital and outpatient respiratory nursing clinic availability 2: variables included in the final patient model: age ≤ 55, gender (male), Charlson index ≥3, number of hospital admissions in the last year ≥1, GesEPOC exacerbator phenotype and being treated in specialized COPD outpatient clinic					

For COPD evaluation category, fulfillment of at least four criteria, the empty model displayed an ICC of 0.30 and a MOR of 3.13 (Table 4). In the adjusted model, an age of ≤ 55 years, FEV1 < 50%, dyspnea ≥ 2 MRC-m and being treated at a specialized COPD outpatient clinic were positively associated with better adherence to CPG recommendations. However, being male and having a Charlson index ≥ 3 were retained as predictors of worse adherence. Some unrecorded values (COPD phenotype missing, dyspnea missing, or level of dyspnea not referred to) showed a significant negative association. Only one variable linked to the hospital level (i.e. respiratory ward availability) was retained as a predictor of better adherence. The inclusion of this predictor further reduced the between-hospital cluster variability. The ICC and MOR dropped to 0.24 and 2.67, respectively.

For therapeutic intervention category, fulfillment of at least three criteria, the empty model displayed an ICC of 0.52 and a MOR of 6.09. A Charlson index ≥ 3, undergoing ≥ 1 hospitalizations in the past year, being treated at a specialized COPD outpatient clinic, and outpatient respiratory nursing clinic availability were associated with better adherence to the recommendations. Meanwhile, being male, being ≤ 55 years old and being a university hospital were all associated with worse adherence. The inclusion of these predictors further reduced the between-hospital cluster variability. The ICC and MOR dropped to 0.44 and 4.74, respectively (Table 4).

## Discussion

The present study constitutes one of the few research papers in the literature that analyze the variability in adherence to current recommendations for COPD clinical practice guidelines for outpatients in Spain. In our analysis, we aimed to study the variables associated with this variability.

This study shows that accounting for the hospital cluster effect, the patient-level and hospital-level predictor variables, partly reduced the unexplained between-hospital variation in adherence. Additionally, it identified a number of variables as predictors of better adherence at the patient and hospital levels. Most predictors were linked to patient characteristics (patient-level) and the type of respiratory clinic in which the patient was treated (general clinic or specialized COPD outpatient clinic).

Being treated at a specialized COPD outpatient clinic was associated with a higher likelihood of adherence to guidelines in the three categories evaluated, and was considered to be of greater importance, compared with the cluster effect, in explaining the between-hospital outcome variations. This is an interesting result, since less than half of the centers had specialized COPD outpatient clinics. In addition, the time available at specialized COPD outpatient clinics to treat the patient was the same as the general outpatient respiratory visit and there was no support nurse. Consequently, this could be considered a proxy for the experience, knowledge and interest of department physicians in the management of COPD patients.

Also, some unrecorded values (COPD phenotype missing and level of dyspnea missing) showed a statistically significant negative association, which are naturally open to interpretation.

The clinical COPD phenotype according to the Spanish National Guideline for COPD (GesEPOC) was collected in 46.3% of the audited patients.

Only 2 (outpatient respiratory nursing clinic and a respiratory ward availability) of the 46 hospital-level variables examined were retained in the model associated with a higher likelihood of implementing CPG recommendations. On the contrary, being a university hospital or large hospital were negatively associated factors. Nevertheless, given the small amount of cluster variability left unexplained in the analysis, it is unlikely that relevant hospital-level variables were not revealed. It’s possible that this finding is the result of a relative small hospital sample size (*N* = 59). Thus, medical care in COPD does not require complex interventions and the majority of respiratory units offered a functional respiratory laboratory. We must consider the fact that this study did not include information about work organization such as COPD clinical management protocol availability or electronic/digital information availability. It also did not include the number of respiratory physicians or respiratory nurses available in the area around the clinic or the professional experience of treating physicians, which might explain a proportion of the total variance due to the center effect.

Our findings are similar to those of previous studies that have demonstrated significant variability in the processes of COPD care. In the European COPD Audit [13], a considerable variability in recommendation guideline suitability was described and only hospital characteristics were related to a minority of indicators. The adherence to guidelines also varied with hospital size, but the differences were small and inconsistent. Previous studies have shown adherence to clinical guidelines was a strong predictor of a favorable outcome. Roberts et al. [11] have suggested that a hospital’s resources are potential components of the unexplained variation in outcomes. A greater number of medical and nursing staff was identified as a protective factor for intra-hospital mortality. In AUDIPOC Spain [12, 20], the large hospital COPD volume and the number of COPD patients admitted to the hospital the year prior to admission was identified as a predictor of a favourable outcome.

In our study, a large component of center-related variance remained unexplained, suggesting that the clinical profile of patients included in the study also varied markedly among hospitals. It is important to remember that recommendation guidelines are evidence-based and aimed to systemize medical care, but the clinical presentation of COPD is variable [21].

Our study has several strengths and limitations. The main strength is its sample size that accounts for 39% of the Spanish population. Nevertheless, the limitations to be considered are the fact that the selection of participating centers was not random and hospital participation was voluntary based on their interest to participate. Also, clinical records were used as the data source, so some missing and inconsistent values were unavoidable. Despite these limitations, we believe that this dataset represents the largest available comparative survey of Spanish centers.

## Conclusions

High variability was observed among hospitals in terms of medical care. Some of the patient’s characteristics (airflow obstruction, degree of dyspnea, exacerbation risk, presence of comorbidities) and the type of respiratory clinic in which the patient was treated (specialized COPD outpatient clinic) were identified as factors associated with a better adherence to recommendations, though a great part of the variability among center cannot be explained. This suggests that there is a significant inconsistency among centers in the implementation of clinical guidelines.

This information must be accounted for by health care professionals and administrators, in order to establish better clinical practice by means of the medical care in the specialized COPD outpatient clinic and the implementation of evidence-based best clinical practice guidelines that could facilitate a uniform approach to COPD patients as outpatients, thereby both improving patient outcomes and optimizing medical resources.

## Figures and Tables

**Fig. 1:**
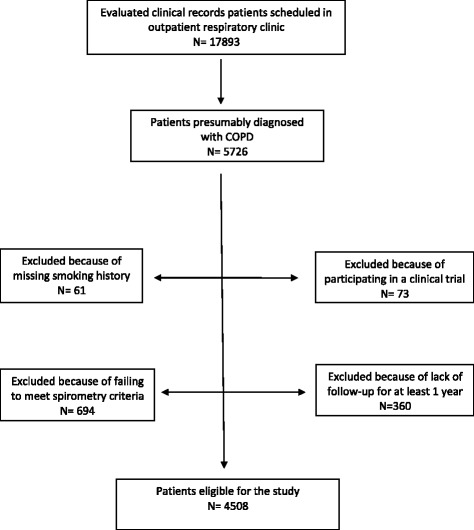
The sampling process is described in a STROBE flow chart

## Appendix 1

**Table 5:** The inclusion criteria and exclusion criteria

The inclusion criteria	- patients aged ≥40 years - smokers or ex-smokers (of at least 10 pack-years) - COPD diagnosed on the basis of spirometric tests (FEV1/FVC post-bronchodilation < 0.7 or FEV1/FVC pre-bronchodilation < 0.7 and FEV1 ≥ 80%, if there is no bronchodilation reversibility testing available
The exclusion criteria	- lack of follow-up for at least 1 year in a respiratory outpatient clinic - participating in a clinical trial

## Appendix 3

**Table 6:** Participating hospitals and catchment population by Autonomous Community

Region of Spain	Number of participating hospitals	Population assigned for admission	Population of the region	Catchment population of the EPOCONSUL study (%)
Andalucía	10	2.784.083	8.424.102	33
Aragón	2	597.000	1.346.293	44.3
Asturias	1	250.000	1.081.487	23.1
Islas Baleares	2	575.000	1.113.114	51.6
País Vasco	4	1.285.000	2.184.606	58.8
Islas Canarias	1	700.000	2.126.769	32.9
Cantabria	2	395.000	593.121	66.6
Castilla y la Mancha	4	1.186.014	2.115.334	56
Castilla y León	4	1.119.086	2.558.463	43.7
Cataluña	5	1.657.000	7.539.618	22
Extremadura	1	273.977	1.109.367	24.7
Galicia	2	970.000	2.795.422	34.7
Madrid	11	3.484.995	6.489.680	53.7
Murcia	3	770.175	1.470.069	52.3
Navarra	1	517.020.	642.051	80.5
Valencia	6	1.540.000	5.117.190	30
TOTAL	59	18.104.350	46 .064 .635	39.3

Data are presented as Numbers. The percentages refer to the total population number

There was no participating hospital in La Rioja, the 17^th^ Autonomous Community in Spain

## Appendix 4

### Participants investigators in EPOCONSUL study

*Andalucía*: Jose Luis Rojas Box, H. de Alta Resolución de Écija, Sevilla. Jose Domingo Garcia Jimenez, H. de Alta Resolución de Utrera, Sevilla. Adolfo Domenech del Rio, Ana Muñoz. H. Carlos Hayas, Málaga. Antonia Soto Venegas, H. San Juan de la Cruz, Úbeda, Jaén. Aurelio Arnedillo Muñoz. H. U. Puerta del Mar, Cádiz. Agustín Valido Morales. H. Virgen de Macarena. Sevilla. Jose Velasco Garrido, Carlos Rueda Ríos, Macarena Arroyo Varela H. Virgen de la Victoria. Málaga. Francisco Ortega Ruiz, Eduardo Marquez Martin, Carmen Calero Acuña, H. Virgen del Rocio, Sevilla. Francisco Luis Garcia Gil, H. U Reina Sofia, Córdoba.

*Aragón*: Joaquin Carlos Costan Galicia, H. Clínico U. Lozano Blesa, Zaragoza. Ana Boldova Loscertales, H. Royo Villanova, Zaragoza.

*Asturias*: Cristina Martinez González, Rosirys Guzman Taveras, H. U. Central de Asturias, Oviedo.

*Murcia*: Juan Luis De la Torre Alvaro, H. U Santa Lucia, Cartagena, Mª Jesus Avilés Ingles, H. General U. Reina Sofia, Murcia. Rubén Andújar Espinosa, H.U. Virgen de la Arrixaca, Murcia.

*Canarias*: Juan Manuel Palmero Tejera, Juan Marco Figueira Conçalves, H.U. Nuestra Señora de la Candelaria, Santa Cruz de Tenerife.

*Cantabria*: Ramon Agüero Balbín, Carlos Amado Diago, Beatriz Abascal Bolado.

H. Marqués de Valdecilla, Santander. Juan Luis Garcia Rivero, Marcelle Cohen Escovar, H. de Laredo, Santander.

*Castilla y la Mancha*: Francisco Javier Callejas González. Complejo hospitalario universitario de Albacete, Albacete. Angel Ortega Gonzalez. H Nuestra Señora del Prado, Talavera de la Reina, Toledo. Rosario Vargas Gonzalez, H. Virgen de la Luz, Cuenca. Encarnación López Gabaldón, Raul Hidalgo Carvajal, H. Virgen de la Salud, Toledo.

*Castilla y León*: Elena Bollo de Miguel, Silvia Fernández Huerga, Complejo Hospitalario Universitario de León. Ana Pueyo Bastida, Complejo Asistencial de Burgos, Burgos. Jesus R Hernández Hernández, Ruth Garcia García, H. Nuestra Señora de Sonsoles, Ávila. Miguel Barrueco Ferrero, Marco López Zuibizarreta, E. Consuelo Fernández, H. Universitario de Salamanca.

*Cataluña*: David De la Rosa Carrillo, H. Plató, Barcelona. Jordi Esplugas Abós, Noelia Pablos Mateos, H. Sant Joan de Déu, Martorell. Elena De Miguel Campos, H. Sant Joan de Despi, Barcelona. Pablo Rubinstein, Hospital General de Cataluña, Barcelona. Hernán Abraham Manrique Chávez, H Sagrat Cor, Barcelona. Miriam Barrecheguren, H. U. Vall d’Hebron, Barcelona.

*Valencia*: Carmen Aguar Benito, H. de Arnau de Villanova, Valencia. Pablo Catalán Serra, H. de Requena, Requena. Eusebi Chiner Vives. H. U. de San Juan, Alicante. Juan Antonio Royo Prats. H. General de Castellón, Castellón de la Plana. Cristina Sabater Abad, Esther Verdejo Mengual, H. General Universitario de Valencia. Eva Martínez- Moragon, H. Universitario Dr. Peset, Valencia.

*Extremadura*: Francisca Lourdes Marquez Perez, H. U Santa Cristina, Badajoz.

*Galicia*: Alberto Fernandez Villar, Cristina Represas Represas, Ana Priegue Carrera, Complejo hospitalario de Vigo. Marina Blanco Aparicio, Pedro Jorge Marcos Rodriguez, H. U. Juan Canalejo, La Coruña.

*Baleares*: Federico Gonzalo Fiorentino, Mª Magdalena Pan Naranjo, H. Son Espases, Palma de Mallorca. Antonia Fuster Gomila, H. Sant Llatzer, Palma de Mallorca.

*Madrid*: German Peces Barba, Felipe Villar Alvarez, Fundación Jimenez Diaz, Madrid. Carlos Jose Álvarez Martinez, H. 12 de Octubre, Madrid. Juan Luis Rodriguez Hermosa, J.L. Álvarez Sala-Walther, Juan Rigüal Bobillo, Gianna Vargas Centanaro, H. Clinico San Carlos, Madrid. José Andrés García Romero de Tejada, H. U. Infanta Sofía, San Sebastián de los Reyes, Madrid. Javier Jareño, Sergio Campos Tellez. H. Central de la Defensa, Madrid. Raul Galera Martinez, H. La Paz. Rosa Mar Gómez Punter, Emma Vázquez Espinosa, H. La Princesa, Madrid. Esther Alonso Peces, H. Principe de Asturias, Alcalá de Henares, Madrid. Juan Manuel Diez Piña, Raquel Pérez Rojo, H. U. de Móstoles, Madrid. Luis Puente Maestu, Julia Garcia de Pedro. H. U. Gregorio Marañón, Madrid. Soledad Alonso Viteri, H. U de Torrejón, Torrejón de Ardoz, Madrid.

*Navarra*: Maria Hernandez Bonaga, Complejo Hospitalario de Navarra, Pamplona.

*País Vasco*: Maria Milagros Iriberri Pascual, H de Cruces, Baracaldo. Myriam Aburto Barrenechea, H de Galdakano. Sophe Garcia Fuika, Hospital Santiago Apostol, Vitoria. Patricia Sobradillo Ecenarro, Hospital Txagorritx, Basurto.

## Appendix 5

**Table 7:** Logistic regression bivariate analysis (adherence to good clinical practice criteria in three categories: clinical evaluation of the patient, COPD evaluation and therapeutic interventions)

Patients	Clinical evaluation ≥ 3 criteria fulfilled OR (95%CI)	*p*	Centers	Clinical evaluation ≥ 3 criteria fulfilled OR (95% CI)	*p*
Age (≤55 years)	1.01 (0.78–1.31)	0.89	Large hospital	0.44 (0.21–0.89)	0.024
Sex (male)	0.96 (0.78–1.17)	0.70	University hospital	0.32 (0.12–0.82)	0.018
Active smokers	1.25 (1.05–1.48)	0.011	Beds per center ≥500	0.57 (0.27–1.22)	0.152
Dyspnea (MRC-m)			Respiratory ward not available	Reference	
0–1			Respiratory ward <20 beds	0.90 (0.24–3.32)	0.876
≥2	Reference		Respiratory ward ≥20 beds	0.51 (0.19–1.35)	0.178
Missing	1.02 (0.83–1.26)	0.797			
Level of dyspnea not referred to	0.24 (0.19–0.31)	<0.001			
	0.11 (0.09–0.15)	<0.001			
FEV1< 50%	1.24 (1.07–1.43)	0.004	Number of pulmonology staff members ≥ 5	0.80 (0.35–1.86)	0.620
Charlson index ≥ 3	1.45 (1.25–1.69)	<0.001	Pulmonology residents present	0.90 (0.41–1.99)	0.806
Number of hospital admissions in the last year ≥ 1	6.45 (5.16–8.07)	<0.001	Number of annual outpatient respiratory visits ≥ 10,000	0.45 (0.17–1.19)	0.109
GesEPOC phenotype			≥ 15 min of follow-up at general outpatient respiratory visit	2.34 (1.13–4.83)	0.021
Non-exacerbator	Reference				
Exacerbator	1.26 (1.00–1.59)	0.048			
Missing	0.40 (0.33–0.49)	<0.001			
Triple inhalation therapy	1.01 (0.87–1.17)	0.872	Specialized COPD outpatient clinic available	1.07 (0.51–2.22)	0.850
Treatment at a specialized COPD outpatient clinic	2.49 (1.91–3.23)	<0.001	Outpatient respiratory nursing clinic availability	2.47 (1.23–4.97)	0.011
			Inhalation technique educational program available	1.61 (0.73–3.55)	0.234
Patients	COPD evaluation ≥4 criteria fulfilled OR (95%CI)	*p*	Centers	COPD evaluation ≥4 criteria fulfilled OR (95%CI)	*p*
Age (≤55 years)	1.68 (1.31–2.14)	<0.001	Large hospital	1.25 (0.66–2.39)	0.484
Sex (male)	0.75 (0.61–0.92)	0.006	University hospital	1.16 (0.49–2.74)	0.729
Active smokers	1.01 (0.82–0.85)	0.824	Beds per center ≥500	1.65 (0.85–3.20)	0.136
Dyspnea (MRC-m)			Respiratory ward not available	Reference	
0–1			Respiratory ward <20 beds	4.23 (1.39–12.86)	0.011
≥2	Reference		Respiratory ward ≥20 beds	2.23 (0.96–5.19)	0.062
Missing	1.53 (1.28–1.84)	<0.001			
Level of dyspnea not referred to	0.51 (0.38–0.68)	<0.001			
	0.54 (0.41–0.69)	<0.001			
FEV1<50%	1.80 (1.55–2.08)	<0.001	Number of pulmonology staff members ≥5	1.44 (0.69–3.00)	0.324
Charlson index ≥3	0.80 (0.69–0.92)	0.003	Pulmonology residents present	1.69 (0.85–3.37)	0.129
Number of hospital admissions in the last year ≥1	1.30 (1.10–1.55)	0.002	Number of annual outpatient respiratory visits ≥10,000	1.82 (0.78–4.25)	0.165
GesEPOC phenotype			≥ 15 min of follow-up at general outpatient respiratory visit	1.06 (0.54–2.09)	0.845
Non-exacerbator	Reference				
Exacerbator	1.38 (1.13–1.69)	0.001			
Missing	0.16 (0.13–0.20)	<0.001			
Triple inhalation therapy	1.37 (1.18–1.60)	<0.001	Specialized COPD outpatient clinic available	1.77 (0.94–3.31)	0.073
Treatment at a specialized COPD outpatient clinic	4.44 (3.48–5.68)	<0.001	Outpatient respiratory nursing clinic availability	1.57 (0.83–2.95)	0.158
			Inhalation technique educational program available	1.91 (0.97–3.75)	0.058
Patients	Therapeutic intervention ≥3 criteria fulfilled OR (95%CI)	*p*	Centers	Therapeutic intervention ≥3 criteria fulfilled OR (95%CI)	*p*
Age (≤55 years)	0.62 (0.44–0.87)	0.007	Large hospital	0.46 (0.16–1.25)	0.130
Sex (male)	0.79 (0.61–1.02)	0.074	University hospital	0.32 (0.08–1.17)	0.087
Active smokers	0.98 (0.80–1.20)	0.894	Beds per centre ≥500	0.78 (0.27–2.25)	0.656
Dyspnea (MRC-m)			Respiratory ward not available	Reference	
0–1	Reference			0.26 (0.04–1.66)	0.158
≥2	1.10 (0.90–1.35)	0.317	Respiratory ward <20 beds	0.32 (0.08–1.19)	0.090
Missing	0.09 (0.05–0.15)	<0.001	Respiratory ward ≥20 beds		
Level of dyspnea not referred to	0.24 (0.17–0.34)	<0.001			
FEV1<50%	1.32 (1.07–1.63)	0.008	Number of pulmonology staff members ≥5	0.41 (0.13–1.27)	0.125
Charlson index ≥3	1.23 (1.04–1.46)	0.016	Pulmonology residents present	0.87 (0.29–2.61)	0.813
Number of hospital admissions in the last year ≥1	1.83 (1.50–2.23)	0.000	Number of annual outpatient respiratory visits ≥10,000	0.29 (0.08–1.06)	0.062
GesEPOC phenotype			≥ 15 min of follow-up at general outpatient respiratory visit	1.09 (0.37–3.20)	0.862
Non-exacerbator	Reference				
Exacerbator	1.10 (0.87–1.38)	0.414			
Missing	0.37 (0.30–0.46)	<0.001			
Triple inhalation therapy	1.02 (0.85–1.21)	0.812	Specialized COPD outpatient clinic available	2.14 (0.79–5.79)	0.133
Treatment at a specialized COPD outpatient clinic	2.95 (2.28–3.83)	<0.001	Outpatient respiratory nursing clinic availability	3.36 (1.27–8.84)	0.014
			Inhalation technique educational program available	3.55 (1.25–10.07)	0.017

## Abbreviations

BMI: Body mass index
COPD: Chronic obstructive pulmonary disease
CPG: Clinical practice guidelines
CSI: Inhaled corticosteroids
GesEPOC: Spanish National Guideline for COPD care
ICC: Intra-cluster correlation coefficient
IHR: Inter-hospital range
IQR: Interquartile range
LABA: Long-acting beta-2 agonists
LAMA: Long-acting antimuscarinic agents
MOR: Median odds ratio
OR: Crude odds ratio
